#  Epidemiologia crítica e saúde coletiva: rupturas e reconstruções 

**DOI:** 10.1590/0102-311XPT086223

**Published:** 2023-08-07

**Authors:** Tarcísio Márcio Magalhães Pinheiro, Fátima Sueli Neto Ribeiro, Adalgisa Peixoto Ribeiro

**Affiliations:** 1 Faculdade de Medicina, Universidade Federal de Minas Gerais, Belo Horizonte, Brasil.; 2 Instituto de Nutrição, Universidade do Estado do Rio de Janeiro, Rio de Janeiro, Brasil.

*Critical Epidemiology and the People’s Health* [Epidemiologia Crítica e a Saúde das Pessoas] ^1^, lançado em 2021, é o terceiro livro editado para a qualificada coleção *Small Books, Big Ideas in Population Health* [Pequenos Livros, Grandes Ideias na Saúde da População], organizada por Nancy Krieger para a Oxford University Press. Essa coleção conta ainda com outras duas obras. Trata-se de uma iniciativa editorial importante e estratégica de divulgação da epidemiologia crítica latino-americana para leitores de língua inglesa. O livro foi publicado originalmente em inglês antes mesmo da edição em espanhol. A epidemiologia crítica emergiu nos anos 1970 no interior do movimento da medicina social latino-americana e um de seus principais formuladores e autores é o professor Jaime Breilh. Protagonista de uma rica trajetória acadêmico-política de mais de 50 anos, Breilh desenvolveu novos conceitos e instrumentos de investigação buscando decifrar as relações entre saúde e sociedade. A epidemiologia crítica vem se consolidando em um novo paradigma, distinto e alternativo aos preceitos da epidemiologia clássica. Sua principal categoria de análise é a determinação social da saúde (DSS). Esse novo livro apresenta uma síntese e reflexão crítica desses vários momentos percorridos pelo autor e pela epidemiologia crítica, em que se percebe o caminhar de um robusto e dialético processo de construção-desconstrução-reconstrução sócio-teórico-metodológico.

A obra em questão é constituída por quatro grandes partes e conta com expressivo e rico conteúdo de referências, além de um providencial e amigável índex, para o deleite do(a) leitor(a).

Na introdução, Breilh apresenta a epidemiologia crítica como “*um ato de busca intelectual crítica compassiva e de resistência audaciosa para se confrontar com um mundo doente*” (p. 1). Como é característico do autor, ficam explicitados seus movimentos, sua postura, sua visão crítica de mundo e alguns de seus valores. São mencionados os limites do conhecimento, as rupturas e revoluções científico-epistemológicas, as relações não neutras das ciências com as sociedades e a complexidade do mundo contemporâneo sob a égide do neoliberalismo. Além disso, realiza uma crítica à epidemiologia cartesiana e propõe formas alternativas teórico-práticas na direção de uma epidemiologia crítica. Essas questões são desenvolvidas e aprofundadas ao longo dos demais capítulos do livro. Frisa-se também que além da profundidade e complexidade de pensamento e conteúdo, a forma pedagógica da exposição é enriquecida com diversos quadros, tabelas e figuras que facilitam o entendimento do(a) leitor(a).

O capítulo 1 é uma bela e didática reconstrução/reflexão do movimento acadêmico-social da saúde coletiva latino-americana desde seus primórdios até os anos recentes. Nele, Breilh mapeia e historiciza as origens, os contextos sociais, políticos, culturais e científicos, os principais autores e os conceitos centrais que deram formato e possibilitaram o processo de desenvolvimento da medicina social/saúde coletiva na região. O autor identifica quatro momentos históricos: nos anos 1970, um enfrentamento ao empirismo causal e ao funcionalismo; nos anos 1980, uma diversificação de métodos e instrumentos de pesquisa; nos anos 1990, uma ênfase na transdisciplinaridade; e nos anos 2000, um movimento rumo à metacrítica intercultural. Às questões sociais, econômicas e políticas são incorporados ao longo do tempo os temas ambientais, de gênero, de raça, de colonização e dos povos originários.

O capítulo 2 retrata a epidemiologia crítica como ciência ousada e ética na busca por compreender uma civilização doentia. Breilh destaca as grandes mudanças decorrentes de um novo estágio do capitalismo neoliberal no século XXI, caracterizado por uma hiperaceleração do capital, com grande concentração de renda, desigualdade social, aumento da precarização do trabalho, informalidade e perda de direitos trabalhistas, além de graves ameaças autoritárias às democracias. Esse macrocenário é detalhado no capítulo 2, com seus impactos epidemiológicos, sanitários, sociais, ideológicos, ambientais, éticos e culturais na saúde e nos sistemas de saúde. Os “deuses” mercado, tecnologia, mídia, consumismo e individualismo são elevados e reforçados com *fake news*. Do ponto de vista epidemiológico, o autor destaca o que se conhece por transição epidemiológica incompleta, coexistindo doenças comuns em populações em situação de vulnerabilidade extrema (desnutrição e agravos infectoparasitários) com doenças de sociedades industriais modernas (neoplasias, distúrbios imunológicos, obesidade e estresse). Breilh questiona o papel da ciência e dos cientistas e advoga uma transformação audaciosa na área da saúde e nas ciências da vida e, mais uma vez, explicita as distinções entre os paradigmas cartesiano e crítico. Discute, ainda, as diferenças entre determinação social e determinismo; reforma radical e reformismo institucional; modo de vida e estilo de vida; bem-estar, bem viver e viver bem; e apresenta os princípios do bem viver ou os quatro “S” da vida: sustentabilidade, soberania, solidariedade e seguridade.

O capítulo 3 propõe uma inovadora matriz-ferramenta de análise e intervenção, que considera o processo histórico-crítico complexo de subsunção que incorpora as dimensões geral (sociedade, movimentos do capital, atores sociais), particular (classe social, gênero, etnia) e individual (estilo de vida, psiquismo), em um movimento dialético, não determinístico, gerando processos protetores e destrutivos para a saúde e a vida. Breilh defende uma nova interpretação, que combina desenhos de pesquisa, métodos quantitativos e qualitativos, em uma perspectiva transdisciplinar, intercultural, ecológica, participativa, decolonial e emancipatória. Essa construção teórico-metodológica é chamada de metacrítica e envolve diversos grupos e movimentos sociais, como sindicatos de trabalhadores, feministas, grupos étnicos, indigenistas, ambientalistas, equipes de defesa dos consumidores, associações comunitárias, entre outros. O autor conclui criticando o tradicional modelo de vigilância epidemiológica cartesiana e propõe o monitoramento participativo (baseado na DSS e incorporando o princípio da precaução), além de alertar sobre a importância da universidade nos processos de repensar e de emancipação social.

Todavia, o paradigma da DSS não é consenso, inclusive no interior da saúde coletiva latino-americana. Atestando essa afirmativa, em 2021, foi publicado um instigante, provocativo, rico e caloroso debate sobre o tema por CSP entre renomados autores a partir de um texto de Minayo ^2^ e robustos comentários de Almeida-Filho ^3^, Breilh ^4^ e Ianni ^5^. Esse debate merece ser lido e relido atentamente. Tal discussão não é nova na saúde coletiva ^6^^,^^7^ e as críticas principais à DSS no texto de Minayo se referem a possíveis vieses do determinismo e à secundarização dos sujeitos/indivíduos. Entendemos que a criteriosa crítica quanto ao “determinismo”/“assujeitismo” não se justifica na obra de Breilh e mesmo no paradigma da DSS. Ao longo do processo de construção e amadurecimento da obra de Breilh, houve sempre uma preocupação de delimitar campos para afastar a DSS do determinismo/estruturalismo. Determinação não é concebida como uma categoria estática, de desfechos inexoráveis e refém do conceito clássico de causalidade. A DSS questiona e supera as concepções fragmentadas de fator de risco e de determinante em saúde. Considera a dimensão social sem negar o particular e o sujeito, numa dinâmica de subsunção, no clássico geral-particular-singular - do micro ao macro e vice-versa, em uma relação dialética. É, pois, um convite ousado e desafiante para se pensar no paradigma de complexidade na saúde coletiva, tanto no entendimento quanto na intervenção na realidade. Há um propósito de se avançar em uma explicação mais robusta, e não ficar apenas na mera descrição descontextualizada, reducionista. A figura do iceberg, frequentemente referida por Breilh, nos apresenta a dimensão do desafio de se evidenciar e ir além do aparente. Considerar e analisar não só o que está acima do nível do mar (emerso), mas sobretudo o que está abaixo (submerso).

A construção da DSS é um processo histórico e se enriquece com esse pequeno/grande livro de Breilh, valioso e imprescindível para os estudiosos e militantes da saúde coletiva e da epidemiologia, em particular para os inseridos na academia, nos serviços de saúde e nos movimentos sociais. Por certo contribuirá muito para reflexão e aprimoramento da práxis em saúde. É uma obra de relevante conteúdo científico-social, citada recentemente na *Lancet* por Horton ^8^, e merece tradução para outros idiomas, de forma a se tornar mais acessível e conhecida.
